# Protective Effects of Astragaloside IV on db/db Mice with Diabetic Retinopathy

**DOI:** 10.1371/journal.pone.0112207

**Published:** 2014-11-20

**Authors:** Yuzhi Ding, Songtao Yuan, Xiaoyi Liu, Pingan Mao, Chen Zhao, Qiong Huang, Rihua Zhang, Yuan Fang, Qinglu Song, Dongqing Yuan, Ping Xie, Yun Liu, Qinghuai Liu

**Affiliations:** 1 Department of Ophthalmology, The First Affiliated Hospital of Nanjing Medical University, Nanjing, China; 2 Department of Ophthalmology, No. 2 People's Hospital of Changzhou, Changzhou, China; 3 Department of Endocrinology, The First Affiliated Hospital of Nanjing Medical University, Nanjing, China; University of Florida, United States of America

## Abstract

**Objectives:**

Diabetic retinopathy (DR) is a common diabetic eye disease which is well-known as the result of microvascular retinal changes. Although the potential biological functions of astragaloside IV (AS IV) have long been described in traditional system of medicine, its protective effect on DR remains unclear. This study aims to investigate the function and mechanism of AS IV on type 2 diabetic db/db mice.

**Methods:**

Db/db mice were treated with AS IV (4.5 mg/kg or 9 mg/kg) or physiological saline by oral gavage for 20 weeks along with db/m mice. In each group, retinal ganglion cell (RGC) function was measured by pattern electroretinogram (ERG) and apoptosis was determined by Terminal deoxynucleotidyl transferase dUTP nick end labeling (TUNEL) staining. Blood and retina aldose reductase (AR) activity were quantified by chemiluminescence analysis. The expressions of phosporylated-ERK1/2, NF-κB were determined by Western blot analysis. Furthermore, the expression of related downstream proteins were quantified by Label-based Mouse Antibody Array.

**Results:**

Administration of AS IV significantly improved the amplitude in pattern ERG and reduced the apoptosis of RGCs.in db/db mice. Furthermore, downregulation of AR activity, ERK1/2 phosphorylation, NF-κB and related cytokine were observed in AS IV treatment group.

**Conclusions:**

Our study indicated that AS IV, as an inhibitor of AR, could prevent the activation of ERK1/2 phosporylation and NF-kB and further relieve the RGCs disfunction in db/db mice with DR. It has provided a basis for investigating the clinical efficacy of AR inhibitors in preventing DR.

## Introduction

Diabetic retinopathy (DR) is known to be the most common microvascular complication of diabetes mellitus and is one of the most serious causes of visual impairment and blindness in developed countries [1]–[3]. Type 1 diabetic patients, along with approximately 80% of insulin-dependent type 2 diabetic patients and 50% of insulin-resistant type 2 diabetic patients, will suffer the retinopathy in the following 20 years since the first diagnosis [4]. Furthermore, DR accounts for 15 to 17% of total blindness [5]. Therefore, there is a pressing need for the development of novel and effective therapeutic approaches to halt the progression of DR. The prime-triggering factor for the progression of the disease is hyperglycemia [6], [7]. However, the pathogenesis remains uncertain. Studies have shown this metabolic disorder can interfere with generation of neurotransmitters [8]–[10], inducing proapoptotic [11]–[14] and proinflammatory responses [15]–[17]. Accumulating evidence also suggested that immunologic and inflammatory mechanisms play important roles in its development and progression [18]. Recently, several vitro and vivo studies indicated that drugs with varying aldose reductase (AR) inhibiting efficacy show significant protection against diabetic complications, which provided evidences pointing to a significant role of AR in mediating hyperglycemic injury [19]–[23].

Astragaloside IV (AS IV), a novel saponin purified from Astragalus membranaceus (Fisch) Bunge, is one of the major and active components of the astragalus membranaceus. It has been reported of its hypoglycemic effect in diabetic mice by inhibiting of hepatic glycogen phosphorylase and glucose-6-phosphatase activities or the formation of advanced glycation end products [24], [25]. Gui et al. suggested AS-IV prevents Glucose-Induced podocyte apoptosis as a novel antioxidant [26]. They also demonstrated significant efficacy against inflammation through inhibiting NF-κB in streptozotocin (STZ)-induced diabetic rats models in diabetic nephropathy (DN) [27]. In addition, AS IV showed its inhibitory effects on diabetic peripheral neuropathy in rats [28]. However, the inhibitory effect of this drug directly on DR has not been explored yet. Based on the observations described above, the aim of the present study was to investigate the protective effects and mechanism of AS IV on diabetic retinopathy in type 2 diabetic db/db mice.

## Research Design and Methods

### Materials

AS IV, extracted from Astragalus membranaceus (Fisch.) Bunge, was provided by the Institute of Botany, Jiangsu Province and Chinese Academy of Sciences (purity >98% using the HPLC method). The rabbit polyclonal antibodies against ERK/p-ERK, NF-κB were purchased from SANTA CRUZ, USA. Terminal Transferase dUTP Nick End Labeling (TUNEL) Assay kit was purchased from Roche, USA. Mouse Cytokine Array was purchased from RayBiotech, USA.

### Experimental design

C57BLKsJ-db/db mice strain (db/db mice for short. Strain name BKS.Cg-Dock7^m^ +/+ Lepr^db^/J and stock number 000642 in Jackson Laboratories) were purchased from Model Animal Research Center of Nanjing University. This study was carried out in strict accordance with the recommendations in the guide for the care and use of animals of the Association for Research in Vision and Ophthalmology (ARVO) and Committee for Animal Experiments of National Center. The protocol was approved by Ethics Committee of Nanjing Medical University. 8-week-old mice were housed in the Experimental Animal Facilities at Nanjing Medical University under specific-pathogen-free (SPF) conditions. Three groups each consisting of 12 male db/db mice along with 12 db/m mice (heterozygotes (Lepr^db/m^) as littermate controls) were randomly assigned. The animals were fed with a normal diet and dosed daily by oral gavage, at 4.5 or 9 mg/kg of AS IV, or an equal amount of physiological saline.

**Figure 1:**
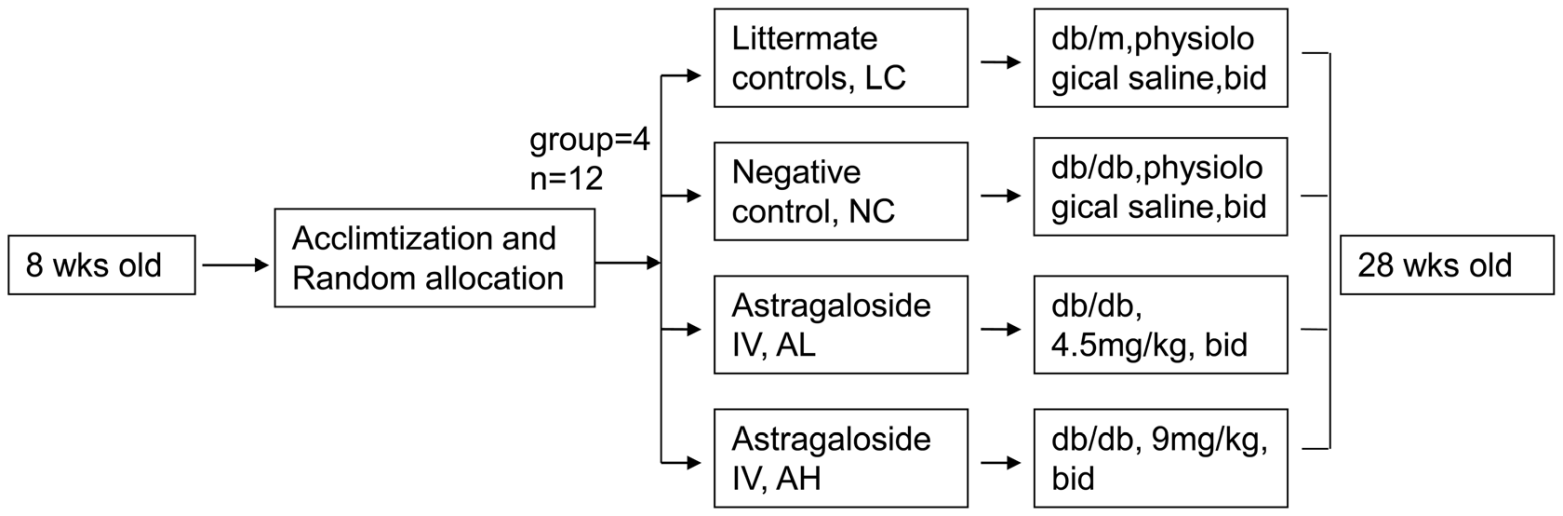
Schematic diagram of experimental design in vivo. 12 db/m mice were treated with physiological saline as a littermate control. Physiological saline was also given to 12 db/db mice as a negative control. 4.5 mg/kg and 9 mg/kg astragaloside IV were treated to see their effects on db/db mice eyes.

Experimental design needed for the present in vivo study has been summarized as follows (Fig. 1):

Group 1 (Littermate controls, LC) consisted of normal db/m animals that received physiological saline only.

Group 2 (Negative control, NC) consisted of db/db mice that received saline orally.

Group 3 (AS IV Low-dose group, AL) consisted of db/db mice that were given As IV orally at a dose of 4.5 mg/kg body weight for 20 weeks.

Group 4 (AS IV High-dose group, AH) consisted of db/db mice that were given AS IV orally at a dose of 9 mg/kg body weight for 20 weeks.

4-weekly body weight measurement and blood glucose monitoring were conducted during the experiment period. The animals were allowed normal access to food and water and were maintained on a 12-h light/dark cycle at controlled temperature (20–25°C) and humidity (50%±5%). All reasonable efforts were made to minimize suffering.

### Electroretinogram Recordings

The pattern ERG has specific potential to detect retinal ganglion cell (RGC) dysfunction or loss [29]. So functional outcomes are needed to follow the progression of db/db mice and assess the therapeutic effects of As IV neuroprotective treatments. We used the amplitude and latency of pattern ERG to monitor progressive RGC dysfunction. After 20 weeks gavage administration, pattern ERGs were recorded from 96 undilated eyes. 36 anesthetized (90 mg/kg ketamine and 6 mg/kg xylazine) db/db mice and 12 anesthetized db/m mice responded to contrast reversal of gratings that maximize pattern ERG amplitude (99% contrast, 1-Hz reversal, spatial frequency of 0.05 cyc/deg). The stimulus display contained four full cycles of the gratings. The center of visual stimulus was aligned with the projection of the pupil. Robust averaging (1800 sweeps) was used to isolate pattern ERG from background noise. The head of anesthetized mice was gently restrained in a modified stereotaxic console, a corneal electrode was placed on the test eye, positioning entailed minimal manipulation of the eye. Reference and ground electrodes were small stainless-steel needles inserted in the skin of the back of the head and the back of the body, respectively. A small drop of balanced saline was added to prevent corneal dryness, and the body of the mouse rested on a feedback controlled heating pad that maintained the body temperature at 37°C. A mouth bite and a small nose holder, which allows unobstructed vision, were used to restrain the anterior part of the head. Under these conditions, the eyes of the mice were naturally proptosed, with undilated pupils pointing laterally and upward. For pattern ERG, response amplitude was evaluated peak-to-trough, and the latency was the time-to-peak of the major positive deflection [29].

### TUNEL Staining

Decreased population of cells is regarded as an important factor in the pathogenesis of various metabolic diseases [30], [31]. To determine the effects of AS IV on RGCs, the apoptosis was determined using TUNEL assays. Only cells marked with green and blue fluorescence can be identified as apoptosis. TUNEL staining was performed according to the manufacturer's protocols to detect diabetic RGC death. After treatment for ERGs recording, animals were sacrificed. Eyes were enucleated and fixed in 4% paraformaldehyde (PFA, wuhan Boster Bio-engineering, Wuhan, China) for 8 h. The eye was cut at the ora serrata and the anterior portion of the eye and lens were removed. The eyecup (retina, choroid, and sclera) was placed in 30% sucrose solution at 4°C overnight for cryoprotection. Eyecups were then embedded in a supporting medium for frozen-tissue specimens (O.C.T. compound, Tissue-Tek, Naperville, IL) and stored in −20°C. The frozen sections will be cut at thicknesses of 8 µm for each sample before use. Slices were collected every 5 pieces and stored at −20°C until labeled. The sections were mounted on glass slides previously treated with poly-L-Lysine. These slides were examined under a laser scanning confocal microscope (LSM710; CarlZeiss, Oberkochen, Germany).

### Retina Aldose reductase (AR) Activity Assay

To assess the reverse effects of AS IV on AR activity in blood and retina, blood sample and retinas were prepared. As for blood sample, AR was isolated from erythrocytes by a modification. The entire procedure was performed at 4°C. About 1 mL of heparinized whole blood obtained from each individual was centrifuged at 1000 rpm for 10 min. After removal of the buffy coat, the erythrocytes were suspended in 2 volumes of PBS and centrifuged again. This washing step was repeated once, and the packed erythrocytes were stored at −70°C until use. Retina complexes were micro-surgically isolated and placed immediately into 200 ul Radio-Immunoprecipitation Assay (RIPA) buffer supplemented with 1 mM protease inhibitor phenylmethane sulfonyl fluoride (PMSF) at 4°C. After mechanical disruption, lysates were placed on ice for 20 minutes and centrifuged at 12,000 rpm for 10 minutes at 4°C. Protein concentrations were determined using a Bradford assay kit (Bio-Rad) with bovine serum albumin as a standard. AR activity was assayed by observing the decrease in the absorbance of NADPH at 367 nm using DL-glyceraldehyde as a substrate [32]. The assay mixture contained 30 mM potassium phosphate buffer (pH 6.5), 5 mM DL-glyceraldehyde (Lot No. 023k2512, Sigma Co.,St.Louis,Mo.), 0.2 M ammonium sulfate, and 1.0 mM β-NADPH (Lot#SLBC6718V, Sigma Co.,St.Louis,Mo.). Fluorescence was measured by fluorescence spectrometer (Thermo Scientific Varioskan Flash Spectral Scanning Multimode Reader, USA) at wavelengths of 367 nm for excitation and 455 nm for emission. One unit of enzyme activity was defined as the amount of enzyme able to oxidize 1 um of NADPH per min at 37°C versus total retina protein.

### Western Blot

To identify possible cellular responses to AS IV in the diabetic model, extracellular signal regulated kinases ERK1/2 and nuclear transcription factor NF-κB were isolated. Following concentration and heating at 100°C for 5 min, 20 µg of protein was run on an SDS-PAGE gel and electro-blotted onto polyvinylidene fluoride membrane (PVDF). The membrane was blocked for 1 h in 5% skim milk in TBST (20 mM Tris-HCl, pH 7.6, 136 mM NaCl, and 0.1% Tween-20) and incubated in the primary antibody at 4°C overnight. After washed 3 times for 5 to 10 min in 50 mL of TBST at room temperature (RT), the membrane was incubated with goat anti-rabbit 1∶2000 for 1 h at RT in 1× TBST, washed 3×10 min, and rinsed with dH_2_O. Detection of the protein was determined by the use of the ECL kit (2 mL/membrane) according to standard procedures. p-ERK/ERK ratio and NF-κB in each group were therefore compared. GAPDH or Histone3 was used as a loading control.

### Mouse Antibody Array-Detection of the Level of inflammatory cytokines and chemokines

Protein expression screening by the Label-based Mouse Antibody Array 1-the differential screening antibody microarray, which was designed and manufactured by RayBiotech, Inc. (Norcross, USA), contains 308 antibodies [33]. Retina protein obtained from db/db or db/m mice were pretreated and resultant supernatant was collected with measured protein contents. Each of the antibodies has two replicates that are printed on a coated glass microscope slide, along with multiple positive and negative controls. The antibody array experiment was performed by Wayen biotechnologies (Shanghai), according to the established protocol. Fluorescence Detection were scanned (GenePix 4000B, Axon Instruments, USA) until glass chips were completely dry and the chips were scanned on a GenePix ×4000 scanner (GenePix 4000B, Axon Instruments, USA) and the images were analyzed with GenePix Pro 6.0 (Axon Inxtruments, USA). After subtracting background signals and normalization to positive controls, comparison of signal intensities between and among array images can be used to determine relative differences in expression levels of each protein between groups. Any ≥1.5-fold increase or ≤0.65-fold decrease in signal intensity for a single analyte between samples or groups may be considered a measurable and significant difference in expression, provided that both sets of signals are well above background.

### Statistical Analysis

The results were expressed as mean ± SD. A comparison between two groups was performed using the two-sided t test and a comparison among multiple groups was subjected to one way analysis of variance (ANOVA) followed by a Kruskal–Wallis Test, with P<0.05 considered statistically significant. Data were analyzed using SPSS 19.0 version.

## Results

### Status of Body Weight and Blood Glucose

**Figure 2:**
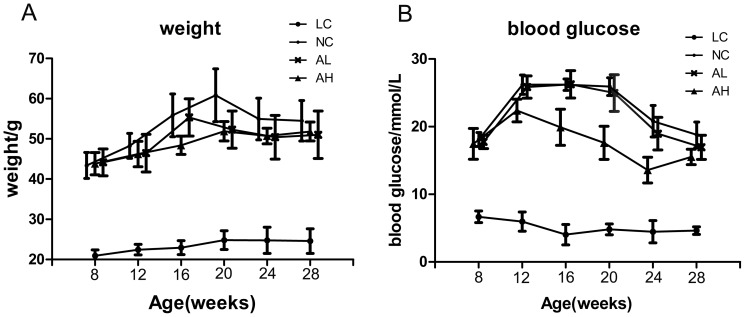
Animal general situations (collected every 4 weeks). A: body weight in all groups during treatment. B: Blood glucose in all groups during treatment.

As can be seen from Fig 2A, mean weights of LC group were less than half of that in db/db mice groups from 8 weeks to 28 weeks. Compared with NC group, AL and AH groups showed less body weight gain from 12 weeks old. The blood glucose levels in NC group were significantly different from that of LC group since 8 weeks. At 8 weeks, there was no difference of glucose levels in all db/db mice groups (Fig. 2B). From 12-week-old to 24-week-old, mice in AH group had decreased blood glucose levels compared with NC group, while at 28-week-old, mice in each db/db mice group showed no difference (AL vs. NC, P = 0.996; AH vs. NC, p = 0.082, respectively). However, in AH group, blood glucose at 28 weeks showed a significant difference compared to baseline (AH: 28 weeks vs. 8 weeks, p<0.01).

### Electroretinograms and TUNEL-Positive Cells

**Figure 3:**
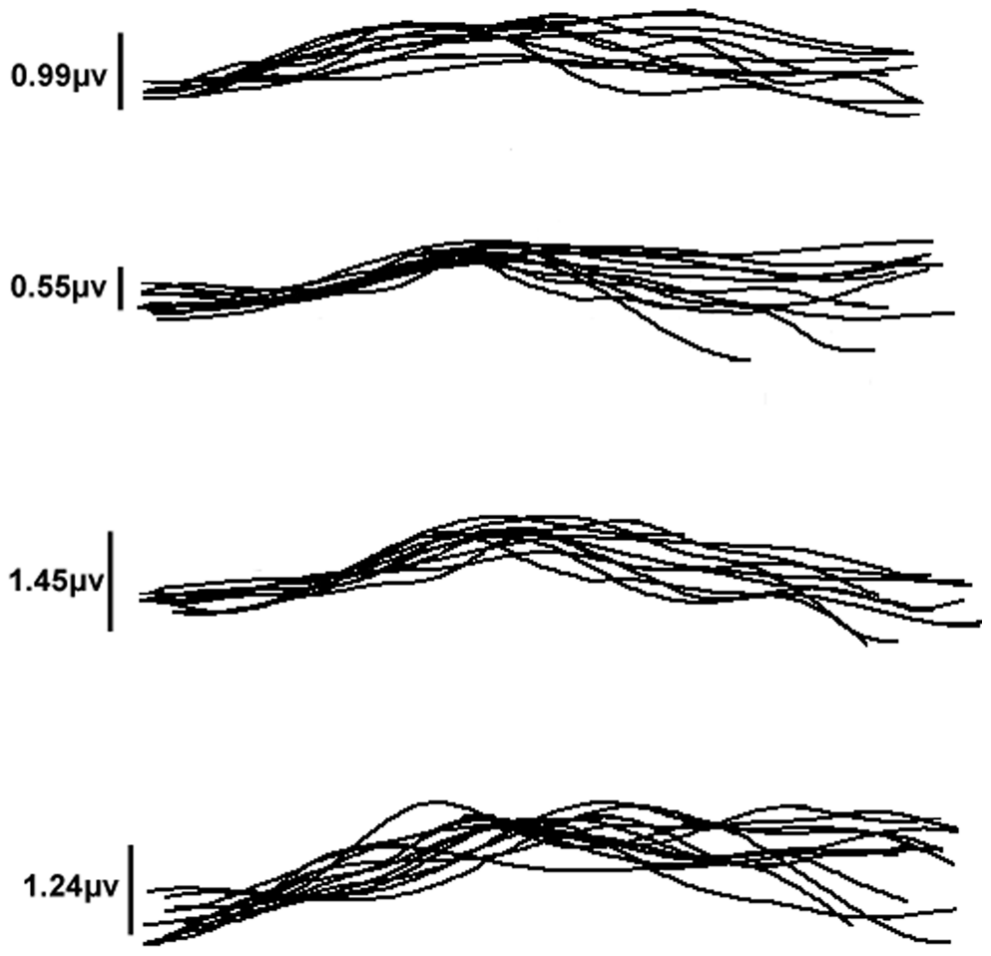
Pattern ERGs derived from each eye in response to pattern reversal of horizontal gratings in a population of 48 mice aged 28 weeks. Individual waveforms of each eye are shown superimposed in each group. Mean amplitude in each group is regarded as a consult. A: LC, B: NC, C: AL, D: AH.

**Figure 4:**
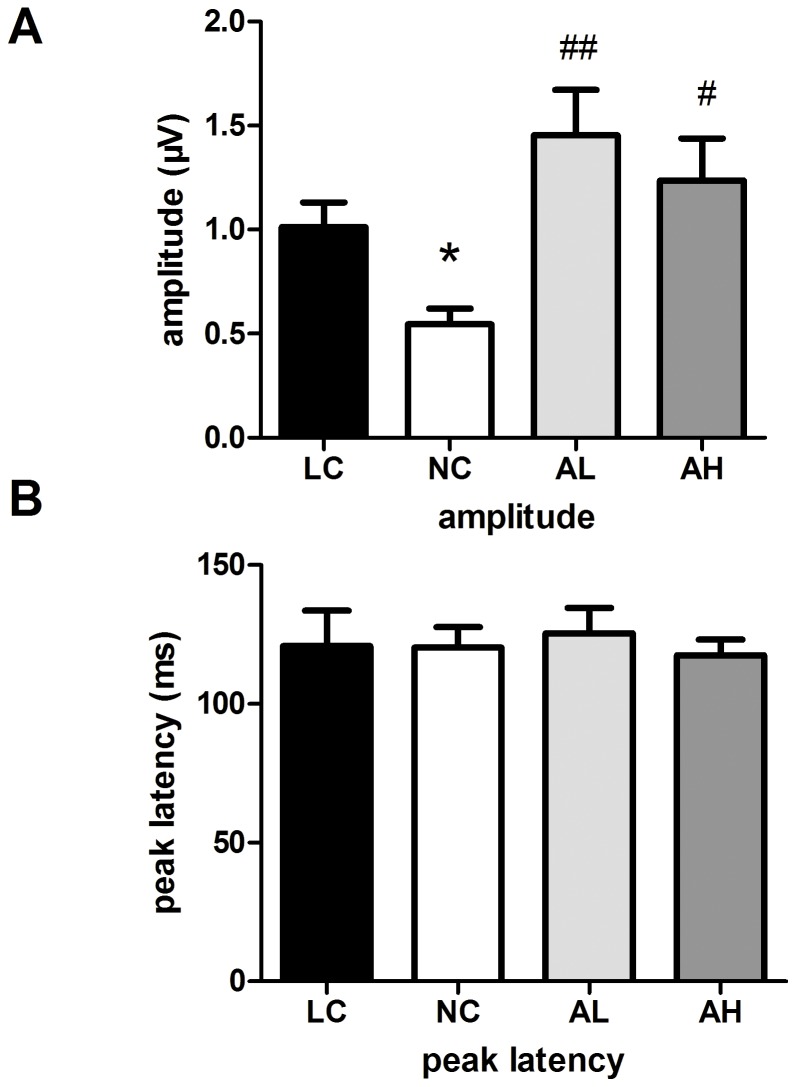
Effects of Astragaloside IV on pattern ERG outcomes. Data are expressed as the mean ± SD. A. Pattern ERG amplitudes in all groups. NC group received significantly decreased amplitudes compared with LC group (NC vs LC, p = 0.037, * p<0.05). Mice in Astragaloside IV treatment groups have increased amplitudes compared with NC group (AL, AH vs NC, p = 0.005, 0.037 respectively. ## p<0.01; # p<0.05). Amplitudes have no significance between AL and AH group. B. Pattern ERG latency in all groups. Astragaloside IV treatment changed the latency, but latency levels did not reveal a statistically significant difference.

**Figure 5:**
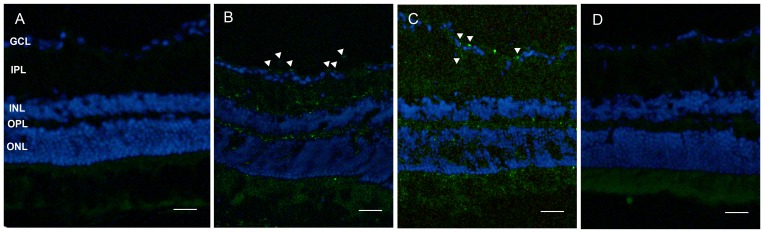
The effect of astragaloside IV on the apoptosis of retina cells. Apoptotic cells were marked with green fluorescence, the nuclei of cells are stained by blue fluorescence (DAPI). A: LC, B: NC, C: AL, D: AH. GCL, retinal ganglion cell layer; IPL, inner plexiform layer; INL, inner nuclear layer; OPL, outer plexiform layer; ONL, outer nuclear layer. Original magnification: ×20; scale bar, 100 mm. Triangles: Apoptotic cells.

Individual waveforms of each eye are shown superimposed in each group (Fig. 3). Fig. 4 shows the measurement of the pattern ERG implicit time and amplitudes at 28 weeks after treatment in the mouse retina. In LC group, the mean amplitude of P_50_ was about 0.99±0.55 µV; the implicit time of P_50_ was about 120.86±33.62 ms. In NC group, the results were changed into 0.55±0.22 µV and 120.33±21.91 ms. In AL and AH groups, the results were changed into 1.45±0.72 µV, 125.45±30.32 ms; 1.24±0.53 µV, 117.50±17.98 ms, respectively. Amplitudes significantly reduced (p = 0.037) in NC group when compared with LC group, indicating the reduced activity of the survival RGCs or the lack of activity of lost RGCs. Amplitudes in AL and AH groups were significantly increased compared with NC group (p = 0.005, 0.037, respectively). AS IV treatment shortened pattern ERG latency, but the change latency levels did not reveal a statistically significance. In Fig. 5, the detection of DNA damage by TUNEL staining indicated that diabetes could induce the apoptosis of RGCs and AS IV decreased the apoptosis of RGCs in retina.

### AR Activity and Responses of Signal Regulators in DR

**Figure 6:**
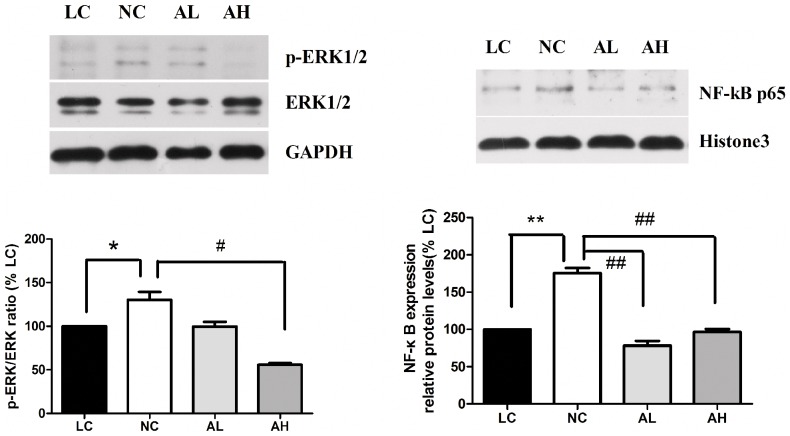
Effects of astragaloside IV on the changes in retina protein. p-ERK/ERK ratio (left) and NF-κB (right) expression in retina protein determined by Western blot. Upper panels are the representative immunoblots of proteins in this area. Bottom graphs show the mean p-ERK/ERK ratio or densitometric quantification of the corresponding bands. GAPDH or Histone3 was used as a loading control. The mean for proteins in db/m mice (LC group) retina was set at 100%. A. p-ERK/ERK ratio: NC vs. LC, p = 0.038. * p<0.05; AL, AH vs NC, p = 0.126, 0.025 respectively. n = 3, # p<0.05). B. NF-κB: NC vs. LC, p = 0.001. ** p<0.01; AL, AH vs. NC, p = 0.001, 0.004 respectively. n = 3, ## p<0.01).

AR activity assay demonstrated a difference between NC group and LC group. AR activity in retina of LC, NC, AL, and AH groups were 17.53±1.12, 22.61±2.29, 20.51±1.58, and 18.07±1.63 U/g protein respectively. Statistic significance was found between LC and NC groups, NC and AH groups (p = 0.026, 0.025, respectively.). AR activity in blood was consistent with that in retina (Table 1). Further analysis of the NF-κB pathway implied the pathogenesis of diabetic retinopathy [34]–[36]. As demonstrated in Fig. 6, NF-κB was significantly upregulated in diabetic retinas compared to LC controls. NF-κB expression in the retinas of NC group was increased by about 75.6% compared to LC group. But in AL and AH groups, the expression were decreased. ERK1/2 phosphorylation was activated in NC group compared with LC group (p = 0.038), while in AH groups, it showed significantly decrease compared with NC group (p = 0.025). The analysis of downstream cytokines of NF-κB and ERK1/2 also showed increased expression of IL-1, IL-6,VEGF,VCAM-1,TNF-βand MMPs in LC compared with NC group (Table 2), but decreased expression in AH compared with NC group.

**Table 1:** Effects of astragaloside IV on dissolve blood Aldose reductase (AR) and retina AR activity (mean with SD) in db/db mice.

Group	N	Blood AR activity (U/g HB)	Retina AR activity (U/g protein)
LC	12	1856.01±61.76	17.53±1.12
NC	12	2192.24±22.25**	22.61±2.29^*^
AL	12	2088.80±37.47^##^	20.51±1.58
AH	12	1845.47±39.46^##^	18.07±1.63^#^

There is a difference between NC and LC group in AR activity (NC vs. LC, p = 0.000, 0.026 in blood AR, retina AR respectively. ** p<0.01; * p<0.05). Astragaloside IV treatment showed a significance in AR activity compared with NC group. (blood AR: AL, AH vs. NC, p = 0.000, 0.000 respectively. Significance also showed in AL and AH groups. p = 0.000. ^##^ p<0.01; retina AR: AL, AH vs. NC, p = 0.218, 0.025 respectively. No significance between AL and AH groups. p = 0.159. ^#^ p<0.05).

**Table 2:** Partial list of related cytokine.

Cytokine	db/m	db/db	db/db + astragaloside IV (9 mg/kg)	ratio1 (NC/LC)	ratio2 (AH/NC)	tendency
IL-1α	2499	4096	2702	1.64	0.65	Down-regulation
IL-1β	1557	2710	1806	1.74	0.67	down-regulation
IL-6	8976	19529	12573	2.18	0.64	down-regulation
VEGF	1029	2241	1131	2.18	0.50	down-regulation
VEGF-D	1397	2236	1433	1.60	0.64	down-regulation
VCAM-1	1893	3083	2016	1.63	0.65	down-regulation
TNF-β	1804	3089	1976	1.71	0.64	down-regulation
MMP-2	1246	2494	1488	2.00	0.60	down-regulation
MMP-3	1273	2361	1471	1.85	0.62	down-regulation
MMP-12	1177	2313	1388	1.97	0.60	down-regulation
MMP-24	2357	4014	2506	1.70	0.62	down-regulation

Cytokine expression changed in db/db mice and reversed by Astragaloside IV.

## Discussion

AS IV has been shown to possess many pharmacological properties, including anti-diabetes [28], antivirus [37], anti-ischemic injury, [38] and anti-inflammation [39]. The anti-inflammatory effect of As IV in DN in STZ-induced diabetic rats has already been reported [27]. The present study demonstrated that AS IV, act as an inhibitor of AR, prevent the activation of ERK1/2 phosporylation and NF-kB, and further relieve the RGCs disfunction in db/db mice with DR.

The db/db mouse is the most widely used mouse model of type 2 diabetes since 1966 [40], especially in DN [41]. As a successful DR model, db/db mouse has been used to investigate the pathogenesis progress in many studies [42]–[46]. Clements used db/db mice to state that Anti-glycated albumin therapy offered prophylaxis against the early changes of diabetic retinopathy [43]. Li used db/db mice to investigate the potential role of lovastatin in diabetic retinopathy in type 2 diabetes [44], [45]. Xiao and Fletcher evaluated retinal neuronal dysfunction in db/db mice [46], [47]. In our study, compared with LC group, higher weight gain and blood glucose levels in NC group indicated the success of this model.

Evidence suggests that retinal neurodegeneration is an early event in the pathogenesis of DR [11], [47]–[49]. Several authors have also reported the presence of retinal neurodegeneration (apoptosis, glial activation and retinal thinning) in db/db mice[50], [51]. Pattern ERG acts as an useful tool to detect the disfunction of RGCs. Recently, Bogdanov et al. [52] have obtained the features of the neurodegenerative process in db/db mice via ERG recording. Our pattern ERG demonstrated that AS IV treatment largely slowed down the dysfunction of RGCs, which is consistent with previous reports. Furthermore, the detection of DNA damage by Tunel staining has also concerned it.

So far, the mechanism of AS IV alleviates diabetes-induced neuronal injury was still unclear. Naeser et al. demonstrated that AR participated in the degeneration of the optic nerve [53], Yu et al. showed inhibitory effects of AS IV on diabetic peripheral neuropathy in rats, which related to the decrease of AR [28]. In the present study, AS IV treatment significantly inhibited activation of AR and the overexpression of NF-κB and ERK1/2 phosphorylation. MAPKs are stress-related kinases, and members of the MAPKs subfamily (JNK, p38, and ERK1/2) have been implicated in neuronal injury and diseases [54]. ERK1/2 is activated by oxidative stress, mitogens, and survival factors, and that regulates cell proliferation and differentiation [55]. Inhibitors of ERKs cooperatively regulated the transactivation potential of the NF-kB p65 subunit [56]. Researches have commented that ERK (MAPK) mediated alterations in early feature of DR [57]. ERK pathway plays a critical role in the induction of processes such as apoptosis. NF-κB is a major transcriptional factor regulating many proinflammatory cytokines, chemokines, and adhesion molecules. Down regulation of IL-1, IL-6, VEGF, VCAM-1, TNF-β and MMPs in our study might linked to the apoptosis of the RGCs.

In conclusion, our data demonstrated the anti-inflammatory and neuroprotective function of AS IV in db/db mice with DR. Therefore, we propose that AS IV, an inhibitor of AR, may act as an potential therapeutic approach for the treatment of DR. Further clinical trials are needed for investigating the clinical efficacy of AS IV.

## References

[1] Madsen-Bouterse SA, Kowluru RA (2008) Oxidative stress and diabetic retinopathy: pathophysiological mechanisms and treatment perspectives. Reviews in endocrine & metabolic disorders 9: 315–327. 18654858 10.1007/s11154-008-9090-4

[2] Antonetti DA, Barber AJ, Bronson SK, Freeman WM, Gardner TW, et al (2006) Diabetic retinopathy: seeing beyond glucose-induced microvascular disease. Diabetes 55: 2401–2411. 16936187 10.2337/db05-1635

[3] Cheung N, Mitchell P, Wong TY (2010) Diabetic retinopathy. Lancet 376: 124–136. 20580421 10.1016/S0140-6736(09)62124-3

[4] Romero-Aroca P, Sagarra-Alamo R, Basora-Gallisa J, Basora-Gallisa T, Baget-Bernaldiz M, et al (2010) Prospective comparison of two methods of screening for diabetic retinopathy by nonmydriatic fundus camera. Clinical ophthalmology 4: 1481–1488. 21191444 10.2147/OPTH.S14521 PMC3009995

[5] Resnikoff S, Pascolini D, Etya'ale D, Kocur I, Pararajasegaram R, et al (2004) Global data on visual impairment in the year 2002. Bulletin of the World Health Organization 82: 844–851. 15640920 PMC2623053

[6] The Diabetes Control and Complications Trial Research Group (1993) The effect of intensive treatment of diabetes on the development and progression of long-term complications in insulin-dependent diabetes mellitus. The New England journal of medicine 329: 977–986. 8366922 10.1056/NEJM199309303291401

[7] UK Prospective Diabetes Study (UKPDS) Group (1998) Intensive blood-glucose control with sulphonylureas or insulin compared with conventional treatment and risk of complications in patients with type 2 diabetes (UKPDS 33). Lancet 352: 837–853. 9742976

[8] Newman EA (2003) New roles for astrocytes: regulation of synaptic transmission. Trends in neurosciences 26: 536–542. 14522146 10.1016/S0166-2236(03)00237-6

[9] Bearse MA Jr, Han Y, Schneck ME, Barez S, Jacobsen C, et al (2004) Local multifocal oscillatory potential abnormalities in diabetes and early diabetic retinopathy. Investigative ophthalmology & visual science 45: 3259–3265. 15326149 10.1167/iovs.04-0308

[10] Han Y, Adams AJ, Bearse MA Jr, Schneck ME (2004) Multifocal electroretinogram and short-wavelength automated perimetry measures in diabetic eyes with little or no retinopathy. Archives of ophthalmology 122: 1809–1815. 15596584 10.1001/archopht.122.12.1809

[11] Barber AJ, Lieth E, Khin SA, Antonetti DA, Buchanan AG, et al (1998) Neural apoptosis in the retina during experimental and human diabetes. Early onset and effect of insulin. The Journal of clinical investigation 102: 783–791. 9710447 10.1172/JCI2425 PMC508941

[12] Abu-El-Asrar AM, Dralands L, Missotten L, Al-Jadaan IA, Geboes K (2004) Expression of apoptosis markers in the retinas of human subjects with diabetes. Investigative ophthalmology & visual science 45: 2760–2766. 15277502 10.1167/iovs.03-1392

[13] Park SH, Park JW, Park SJ, Kim KY, Chung JW, et al (2003) Apoptotic death of photoreceptors in the streptozotocin-induced diabetic rat retina. Diabetologia 46: 1260–1268. 12898017 10.1007/s00125-003-1177-6

[14] Zeng XX, Ng YK, Ling EA (2000) Neuronal and microglial response in the retina of streptozotocin-induced diabetic rats. Visual neuroscience 17: 463–471. 10910112 10.1017/s0952523800173122

[15] Krady JK, Basu A, Allen CM, Xu Y, LaNoue KF, et al (2005) Minocycline reduces proinflammatory cytokine expression, microglial activation, and caspase-3 activation in a rodent model of diabetic retinopathy. Diabetes 54: 1559–1565. 15855346 10.2337/diabetes.54.5.1559

[16] Zhang J, Gerhardinger C, Lorenzi M (2002) Early complement activation and decreased levels of glycosylphosphatidylinositol-anchored complement inhibitors in human and experimental diabetic retinopathy. Diabetes 51: 3499–3504. 12453906 10.2337/diabetes.51.12.3499

[17] Gerhardinger C, Costa MB, Coulombe MC, Toth I, Hoehn T, et al (2005) Expression of acute-phase response proteins in retinal Muller cells in diabetes. Investigative ophthalmology & visual science 46: 349–357. 15623795 10.1167/iovs.04-0860

[18] Tang J, Kern TS (2011) Inflammation in diabetic retinopathy. Progress in retinal and eye research 30: 343–358. 21635964 10.1016/j.preteyeres.2011.05.002 PMC3433044

[19] Kuzumoto Y1, Kusunoki S, Kato N, Kihara M, Low PA (2006) Effect of the aldose reductase inhibitor fidarestat on experimental diabetic neuropathy in the rat. Diabetologia 49: 3085–3093. 17063327 10.1007/s00125-006-0400-7

[20] Tiwari AK, Kumar DA, Sweeya PS, Chauhan HA, Lavanya V, et al (2014) Vegetables' juice influences polyol pathway by multiple mechanisms in favour of reducing development of oxidative stress and resultant diabetic complications. Pharmacognosy Magazine 10: 383–391. 10.4103/0973-1296.133290 PMC4078340 24991118

[21] Chang KC, Ponder J, Labarbera DV, Petrash JM (2014) Aldose reductase inhibition prevents endotoxin-induced inflammatory responses in retinal microglia. Investigative ophthalmology & visual science 55: 2853–2861. 24677107 10.1167/iovs.13-13487 PMC4010364

[22] Noh HL, Hu Y, Park TS, DiCioccio T, Nichols AJ, Okajima K, et al (2009) Regulation of plasma fructose and mortality in mice by the aldose reductase inhibitor lidorestat. journal of pharmacology and experimental therapeutics 328: 496–503. 18974362 10.1124/jpet.108.136283 PMC2682276

[23] Sango K, Suzuki T, Yanagisawa H, Takaku S, Hirooka H, et al (2006) High glucose-induced activation of the polyol pathway and changes of gene expression profiles in immortalized adult mouse Schwann cells IMS32. Journal of neurochemistry 98: 446–458. 16805838 10.1111/j.1471-4159.2006.03885.x

[24] Lv L, Wu SY, Wang GF, Zhang JJ, Pang JX, et al (2010) Effect of astragaloside IV on hepatic glucose-regulating enzymes in diabetic mice induced by a high-fat diet and streptozotocin. Phytotherapy research: PTR 24: 219–224. 19610026 10.1002/ptr.2915

[25] Motomura K, Fujiwara Y, Kiyota N, Tsurushima K, Takeya M, et al (2009) Astragalosides isolated from the root of astragalus radix inhibit the formation of advanced glycation end products. Journal of agricultural and food chemistry 7: 7666–7672. 10.1021/jf9007168 19681610

[26] Gui D, Guo Y, Wang F, Liu W, Chen J, et al (2012) (2012) Astragaloside IV, a Novel Antioxidant, Prevents Glucose Induced Podocyte Apoptosis In Vitro and In Vivo. PLoS One 7(6): e39824. 22745830 10.1371/journal.pone.0039824 PMC3382154

[27] Gui D, Huang J, Guo Y, Chen J, Chen Y, et al (2013) Astragaloside IV ameliorates renal injury in streptozotocin-induced diabetic rats through inhibiting NF-kappaB-mediated inflammatory genes expression. Cytokine 2013 61: 970–977. 10.1016/j.cyto.2013.01.008 23434274

[28] Yu J, Zhang Y, Sun S, Shen J, Qiu J, et al (2006) Inhibitory effects of astragaloside IV on diabetic peripheral neuropathy in rats. Canadian journal of physiology and pharmacology 84: 579–587. 16900242 10.1139/y06-015

[29] Porciatti V, Saleh M, Nagaraju M (2007) The pattern electroretinogram as a tool to monitor progressive retinal ganglion cell dysfunction in the DBA/2J mouse model of glaucoma. Investigative ophthalmology & visual science 48: 745–751. 17251473 10.1167/iovs.06-0733 PMC1794678

[30] Li GY, Fan B, Su GF (2009) Acute energy reduction induces caspase-dependent apoptosis and activates p53 in retinal ganglion cells (RGC-5). Experimental eye research 89: 581–589. 19524568 10.1016/j.exer.2009.06.004

[31] Ji Y, Lu G, Chen G, Huang B, Zhang X, et al (2011) Microcystin-LR Induces Apoptosis via NF-kappaB/iNOS Pathway in INS-1 Cells. International journal of molecular sciences 12: 4722–4734. 21845107 10.3390/ijms12074722 PMC3155380

[32] Yin X, Zhang Y, Wu H, Zhu X, Zheng X, et al (2004) Protective effects of Astragalus saponin I on early stage of diabetic nephropathy in rats. Journal of pharmacological sciences 95: 256–266. 15215651 10.1254/jphs.fp0030597

[33] Huang R, Jiang W, Yang J, Mao YQ, Zhang Y, et al (2010) A biotin label-based antibody array for high-content profiling of protein expression. Cancer genomics & proteomics 7: 129–141. 20551245

[34] Dong N, Chang L, Wang B, Chu L (2014) Retinal neuronal MCP-1 induced by AGEs stimulates TNF-α expression in rat microglia via p38, ERK, and NF-κB pathways. Mol Vis 20: 616–628. 24826069 PMC4016805

[35] Basavarajappa HD, Lee B, Fei X, Lim D, Callaghan B, et al (2014) Synthesis and mechanistic studies of a novel homoisoflavanone inhibitor of endothelial cell growth. PLoS One. 2014 Apr 21 9(4): e95694. 10.1371/journal.pone.0095694 PMC3994091 24752613

[36] Kim J, Kim CS, Sohn E, Lee YM, Jo K, et al (2014) Aminoguanidine protects against apoptosis of retinal ganglion cells in Zucker diabetic fatty rats. Eur Rev Med Pharmacol Sci. 18: 1573–1578. 24943965

[37] Wang S, Li J, Huang H, Gao W, Zhuang C, et al (2009) Anti-hepatitis B virus activities of astragaloside IV isolated from radix Astragali. Biological & pharmaceutical bulletin 32: 132–135. 19122295 10.1248/bpb.32.132

[38] Luo Y, Qin Z, Hong Z, Zhang X, Ding D, et al (2004) Astragaloside IV protects against ischemic brain injury in a murine model of transient focal ischemia. Neuroscience letters 363: 218–223. 15182947 10.1016/j.neulet.2004.03.036

[39] Zhang WJ, Hufnagl P, Binder BR, Wojta J (2003) Antiinflammatory activity of astragaloside IV is mediated by inhibition of NF-kappaB activation and adhesion molecule expression. Thrombosis and haemostasis 90: 904–914. 14597987 10.1160/TH03-03-0136

[40] Hummel KP, Dickie MM, Coleman DL (1966) Diabetes, a new mutation in the mouse. Science 153: 1127–1128. 5918576 10.1126/science.153.3740.1127

[41] Tesch GH, Lim AK (2011) Recent insights into diabetic renal injury from the db/db mouse model of type 2 diabetic nephropathy. American journal of physiology Renal physiology 300: 301–310. 10.1152/ajprenal.00607.2010 21147843

[42] Robinson R, Barathi VA, Chaurasia SS, Wong TY, Kern TS (2012) Update on animal models of diabetic retinopathy: from molecular approaches to mice and higher mammals. Dis Model Mech 5: 444–456. 22730475 10.1242/dmm.009597 PMC3380708

[43] Clements RS Jr, Robison WG Jr, Cohen MP (1998) Anti-glycated albumin therapy ameliorates early retinal microvascular pathology in db/db mice. J Diabetes Complications 12: 28–33. 9442812 10.1016/s1056-8727(97)00051-2

[44] Li J, Wang JJ, Chen D, Mott R, Yu Q, et al (2009) Systemic administration of HMG-CoA reductase inhibitor protects the blood-retinal barrier and ameliorates retinal inflammation in type 2 diabetes. Exp Eye Res 89: 71–78. 19254713 10.1016/j.exer.2009.02.013 PMC2692761

[45] Li J, Wang JJ, Yu Q, Chen K, Mahadev K, et al (2010) Inhibition of reactive oxygen species by Lovastatin downregulates vascular endothelial growth factor expression and ameliorates blood-retinal barrier breakdown in db/db mice: role of NADPH oxidase 4. Diabetes 59: 1528–1538. 20332345 10.2337/db09-1057 PMC2874715

[46] Xiao C, He M, Nan Y, Zhang D, Chen B, et al (2012) Physiological effects of superoxide dismutase on altered visual function of retinal ganglion cells in db/db mice. PLoS One 7: e30343. 22272340 10.1371/journal.pone.0030343 PMC3260298

[47] Fletcher EL, Phipps JA, Ward MM, Puthussery T, Wilkinson-Berka JL, et al (2007) Neuronal and glial cell abnormality as predictors of progression of diabetic retinopathy. Curr Pharm Des 13: 2699–2712. 17897014 10.2174/138161207781662920

[48] Barber AJ, Gardner TW, Abcouwer SF (2011) The significance of vascular and neural apoptosis to the pathology of diabetic retinopathy. Invest Ophthalmol Vis Sci 52: 1156–1163. 21357409 10.1167/iovs.10-6293 PMC3053099

[49] Antonetti DA, Klein R, Gardner TW (2012) Diabetic retinopathy. N Engl J Med 366: 1227–1239. 22455417 10.1056/NEJMra1005073

[50] Cheung AK, Fung MK, Lo AC, Lam TT, So KF, et al (2005) Aldose reductase deficiency prevents diabetes-induced blood-retinal barrier breakdown, apoptosis, and glial reactivation in the retina of db/db mice. Diabetes 54: 3119–3125. 16249434 10.2337/diabetes.54.11.3119

[51] Tang L, Zhang Y, Jiang Y, Willard L, Ortiz E, et al (2011) Dietary wolfberry ameliorates retinal structure abnormalities in db/db mice at the early stage of diabetes. Exp Biol Med (Maywood) 236: 1051–1063. 21750018 10.1258/ebm.2011.010400 PMC3166358

[52] Bogdanov P, Corraliza L, Villena JA, Carvalho AR, Garcia-Arumí J, et al (2014) The db/db mouse: a useful model for the study of diabetic retinal neurodegeneration. PLoS One 9: e97302. 24837086 10.1371/journal.pone.0097302 PMC4023966

[53] Naeser P, Brolin SE, Berggren PO (1987) The sorbitol shunt in the retina and the optic nerve of mice with inherited and STZ-induced diabetes. Acta Ophthalmol (Copenh) 65: 693–698. 3434235 10.1111/j.1755-3768.1987.tb07065.x

[54] Miloso M, Scuteri A, Foudah D, Tredici G (2008) MAPKs as mediators of cell fate determination: an approach to neurodegenerative diseases. Curr Med Chem 15: 538–548. 18336268 10.2174/092986708783769731

[55] Luo Y, DeFranco DB (2006) Opposing roles for ERK1/2 in neuronal oxidative toxicity: distinct mechanisms of ERK1/2 action at early versus late phases of oxidative stress. J Biol Chem 281: 16436–16442. 16621802 10.1074/jbc.M512430200

[56] Vanden-Berghe W, Plaisance S, Boone E, De Bosscher K, Schmitz ML, et al (1995) P38 and extracellular signal-regulated kinase mitogen-activated protein kinase pathways are required for nuclear factor- kB p65 transactivation mediated by tumor necrosis factor. Science 270: 2008–2011. 9452444 10.1074/jbc.273.6.3285

[57] Kim JH, Kim JH, Jun HO, Yu YS, Kim KW (2010) Inhibition of protein kinase C delta attenuates blood-retinal barrier breakdown in diabetic retinopathy. Am J Pathol 176: 1517–1524. 20110406 10.2353/ajpath.2010.090398 PMC2832170

